# Prevalence of test anxiety among medical students: a systematic review and meta-analysis

**DOI:** 10.1186/s12909-026-10270-2

**Published:** 2026-09-09

**Authors:** Yusof Mohamed Omar, Nour Edin Darwish, Mohamed Ashraf Elsaadany, Yousuf Darwish, Youssef Eladawy

**Affiliations:** 1 Faculty of Medicine, Mansoura University, Mansoura, Egypt; 2 Faculty of Medicine, Cairo University, Cairo, Egypt

**Keywords:** Test anxiety, Exam anxiety, Prevalence, Medical students, Systematic review, Meta-analysis

## Abstract

**Background:**

Test anxiety is a situation-specific construct that can markedly impair cognitive functioning and academic performance in high-stakes educational environments. Medical students are frequently exposed to evaluative situations, and test anxiety is common in this population; however, reported prevalence estimates vary widely due to methodological inconsistencies across studies. Therefore, we aimed to estimate the pooled prevalence of test anxiety among medical students.

**Methods:**

We searched PubMed, Scopus, Embase, and Web of Science in March 2026, supplemented by backward and forward citation searching. Studies were eligible if they quantitatively assessed test anxiety among medical students and reported a discrete prevalence estimate. The pooled prevalence was calculated using a random-effects model with a generalized linear mixed model and logit transformation. Subgroup analyses were conducted by gender, academic year, geographic region, assessment tool, administration timing, and sampling technique. A meta-regression examined the association between publication year and prevalence. Risk of bias was assessed using the Newcastle-Ottawa Scale adapted for descriptive cross-sectional studies (NOS-xs2).

**Results:**

Thirty-five studies comprising 13,489 medical students were included. The overall pooled prevalence of test anxiety was 49.8% (95% CI: 39.9%–59.6%, I² = 98.2%, Tau² = 1.33), which decreased to 44.9% upon exclusion of studies utilizing the Visual Analogue Scale (*n* = 3). Among subgroup analyses, only the assessment tool showed a significant effect. Nevertheless, prevalence was numerically higher in females (52.6%) than males (46.3%), among first-year students (58.6%) compared with fifth-year students (47.9%); and highest in South-East Asia (59.3%), followed by the Middle East (54.1%). Prevalence was also highest in studies conducted during examinations (55.4%), compared with pre-examination (51.7%) and post-examination (41.5%) periods, and in studies using convenience sampling (52.9%) compared to random sampling (44.7%). Meta-regression showed no significant association between publication year and prevalence (β = 0.003, *p* > 0.9).

**Conclusions:**

Test anxiety affects nearly half of all medical students globally. The substantial heterogeneity underscores the need for standardised measurement and precise reporting of administration timing in future research.

**Supplementary Information:**

The online version contains supplementary material available at https://doi.org/10.1186/s12909-026-10270-2.

## Introduction

Medical education is inherently characterized by rigorous academic demands and frequent high-stakes evaluations. These assessments often have significant consequences for students’ professional progression and are therefore associated with substantial anticipatory distress in the period preceding examinations [1]. Test anxiety, also referred to as exam anxiety, is a situation-specific, multidimensional construct comprising cognitive components and physiological autonomic arousal triggered by the anticipated evaluative threat [2]. These cognitive components include catastrophic worry and intrusive thoughts, while the physiological arousal may manifest as increased heart rate, muscle tension, rapid breathing, and gastrointestinal discomfort. Unlike general trait anxiety which reflects a chronic tendency to perceive threats [3], test anxiety is a temporary but intense emotional state that can markedly impair cognitive functioning [4].

Although mild test anxiety may be beneficial and has been reported to enhance focus and academic performance [5, 6], excessive test anxiety can overwhelm cognitive resources and impair learning and exam performance [4]. Theoretical frameworks suggest that this occurs because test anxiety consumes students’ working memory resources, dividing attention between task demands and self-deprecating thoughts [7, 8]. This reduction in available cognitive capacity slows information processing and can lead to underperformance that does not accurately reflect a student’s true competency. This poor performance, in turn, reinforces feelings of inadequacy and self-doubt, creating a cyclical pattern in which anxiety further undermines confidence and academic outcomes [9]. This issue is especially pronounced in medical education, where frequent high-stakes assessments place considerable pressure on students and heighten the impact of excessive test anxiety [10].

Despite the significant burden of test anxiety on medical students, the reported prevalence of test anxiety varies widely in the literature, with estimates ranging from as low as 10.7% [11] to as high as 99% [12]. This heterogeneity is partly attributable to methodological inconsistencies. For instance, studies vary widely in the timing of assessment—conducted before [13], after [14], or even during examinations [15], with some failing to report timing altogether [16–18]—resulting in substantial variability in reported prevalence estimates. In addition, definitions of cases of test anxiety differ considerably across studies, with some studies classifying moderate anxiety as pathological [17], whereas others restrict case definitions to high or severe anxiety only [11, 19]. Moreover, a subset of studies relies on relative sample-based splits rather than established cutoffs [20, 21]. As noted by Cassady et al. [22], this approach artificially constrains prevalence estimates to reflect sample distribution characteristics rather than true clinical burden, potentially obscuring the magnitude of the problem and masking the influence of study-level moderators.

Previous systematic reviews on test anxiety among medical students have focused on specific contexts, such as anxiety during objective structured clinical examinations [23] or its association with academic performance [24]. Furthermore, although recent meta-analysis by Ansari and Iqbal [25] estimated the overall prevalence of test anxiety at approximately 48% in the general student population and 47.3% among healthcare students, this estimate aggregates data from medical, nursing, and allied health programs, failing to isolate the specific burden associated with the distinctively high-stakes environment of medical training. Given the substantial impact of test anxiety on medical students and the marked methodological heterogeneity in prevalence reporting, we aimed to estimate the global prevalence of test anxiety specifically among medical students, thereby providing a more reliable evidence base to inform educational policy and student support services.

## Methods

We followed the recommendations of the Preferred Reporting Items for Systematic reviews and Meta-Analyses (PRISMA) guidelines while reporting this manuscript [26]. This study was prospectively registered on PROSPERO with the registration number CRD420261347431. The review question was structured using a modified PE(O) framework, consisting of the Population (medical students) and Outcome (test anxiety), while associated factors (Exposure (E)) reported across studies were synthesized descriptively.

### Literature search strategy

We searched PubMed, Scopus, Embase, and Web of Science in March 2026. Our search strategy included synonyms for test anxiety (e.g. exam anxiety) and medical students (e.g., medical trainees, health profession students). The query was adapted for each database, and the full search strategy is provided in Table S1 (Supplementary Material 1). In addition, we performed backward citation searching by screening the reference lists of all included studies, as well as a recent systematic review and meta-analysis of test anxiety among mixed student populations to identify additional relevant studies [25]. Following backward citation searching, we conducted forward citation searching by screening the studies that cited the included articles.

### Eligibility criteria and outcome definition

We included all studies that quantitatively assessed test anxiety among undergraduate medical students using any test anxiety measurement tool, such as, but not limited to, the Westside Test Anxiety Scale (WTAS) [27], Sarason’s Test Anxiety Scale (TAS) [2], and the Test Anxiety Questionnaire (TAQ) [28].

We harmonized severity classifications across studies, defining test anxiety cases as those meeting moderate, high, or severe/extreme thresholds. For example, students were counted as cases if they fell in the “moderately high” to “extremely high” categories on the WTAS or in the “moderate” to “severe” categories on the TAS. When studies merged low and moderate levels into a single category, only 50% of participants in that category were included to provide a conservative estimate and minimize potential overestimation of clinically significant test anxiety prevalence. In studies that explicitly framed the moderate tier as “healthy” or “facilitating” anxiety (e.g., Nist and Diehl’s healthy anxiety), we restricted case classification to the high or severe categories to avoid misclassifying adaptive anxiety as pathological anxiety.

We excluded studies that reported test anxiety solely as continuous scores without applying cut-off points, as well as those that defined anxiety levels using local sample splits (e.g., median, mean, or quartile splits). Authors reporting test anxiety as continuous scores were not contacted. Consistent with the recommendations of Cassady et al. [22], using local sample splits merely identifies the most test-anxious individuals within a given sample rather than those meeting a fixed threshold, rendering prevalence estimates fixed (e.g., 25% or 50%) and not comparable across populations. Studies were also excluded if they assessed test anxiety through self-identification or qualitative methods. We also excluded studies on non-medical student populations (e.g., nursing, dental students, or interns) or graduate medical students (interns and residents), as well as those that reused the same sample for different analyses (e.g., factor analysis). Reviews and editorials were also excluded.

### Selection process and data extraction

Eligibility screening was conducted in two stages. First, titles and abstracts of all retrieved papers were screened using Rayyan software [29]. Next, the full texts of eligible studies were reviewed to determine their eligibility for inclusion in the review. Two authors independently conducted the screening and selection process, with any disagreements resolved through consultation with a third author.

Four authors independently extracted data using Google Sheets, with an independent fifth author reviewing the data to resolve discrepancies. The extracted data included study design characteristics, population details, assessment tools used, survey administration timing, cutoff scores employed, reported prevalence of test anxiety, and key study findings. For studies employing longitudinal or repeated-measures designs, data were extracted specifically from the pre-examination time point to accurately capture state anxiety triggered by evaluative pressure.

### Risk of bias

Two authors independently assessed the risk of bias of the included studies. Although the dataset included studies with varying designs (cross-sectional, longitudinal, and interventional), only baseline data were extracted for the meta-analysis of prevalence. Accordingly, all studies were evaluated using the recently adapted simplified version of the Newcastle-Ottawa Scale (NOS-xs2) adapted for descriptive cross-sectional (prevalence) studies [30]. Specifically, these domains are assessed through three items: representativeness of the sample, sample size, and assessment of the outcome.

### Statistical analysis

All statistical analyses were performed using R [31] with the meta [32] and metafor [33] packages.

The overall pooled prevalence of test anxiety was calculated using a random-effects model, employing a generalized linear mixed model (GLMM) with a logit transformation for pooling. Confidence intervals for the summary estimates were calculated using a t-distribution with k-1 degrees of freedom, an approach analogous to the Knapp-Hartung adjustment for GLMMs. Additionally, 95% prediction intervals (PIs) were calculated.

Heterogeneity among the included studies was primarily evaluated and quantified using the absolute between-study variance (Tau²), which was estimated using the maximum-likelihood estimator. Because I² estimates are artificially inflated in prevalence meta-analyses with large sample sizes (high precision), we primarily relied on Tau² to quantify heterogeneity [34].

Subgroup analyses were conducted based on categorical variables, including gender, academic year, geographical region, assessment tool, administration timing, and sampling technique. For these subgroup analyses, a common Tau² was assumed across subgroups. Furthermore, a meta-regression was conducted to examine the potential association between data collection year and the reported prevalence of test anxiety. A p-value of less than 0.05 was considered statistically significant.

A leave-one-out sensitivity analysis was performed to evaluate the robustness of the overall pooled estimate and to identify whether any single study disproportionately influenced the summary prevalence or the overall degree of heterogeneity. An additional sensitivity analysis was conducted excluding studies that used the Visual Analogue Scale (VAS), the only non-validated instrument among the tools used in the included studies. A further sensitivity analysis was also conducted for studies in which low and moderate anxiety levels were merged into a single category and 50% of that category was included in the main analysis; in this analysis, only participants classified within the severe/high anxiety categories were included.

Publication bias was not formally assessed in this meta-analysis. Standard procedures for detecting publication bias, such as funnel plot asymmetry, are based on the premise that statistical significance influences publication likelihood. However, this framework is conceptually misaligned with prevalence studies, where researchers report epidemiological proportions rather than assessing significant effects or relationships against specific values. Therefore, applying standard publication bias procedures to prevalence data is considered inappropriate [35].

## Results

### Search results

Our database search retrieved 1,101 records, of which 243 were duplicates. After duplicate removal, 858 records remained for title and abstract screening, during which 740 were excluded. A total of 120 full-text articles were assessed for eligibility, of which 28 met the inclusion criteria by reporting the discrete prevalence of test anxiety among medical students.

**Fig. 1:**
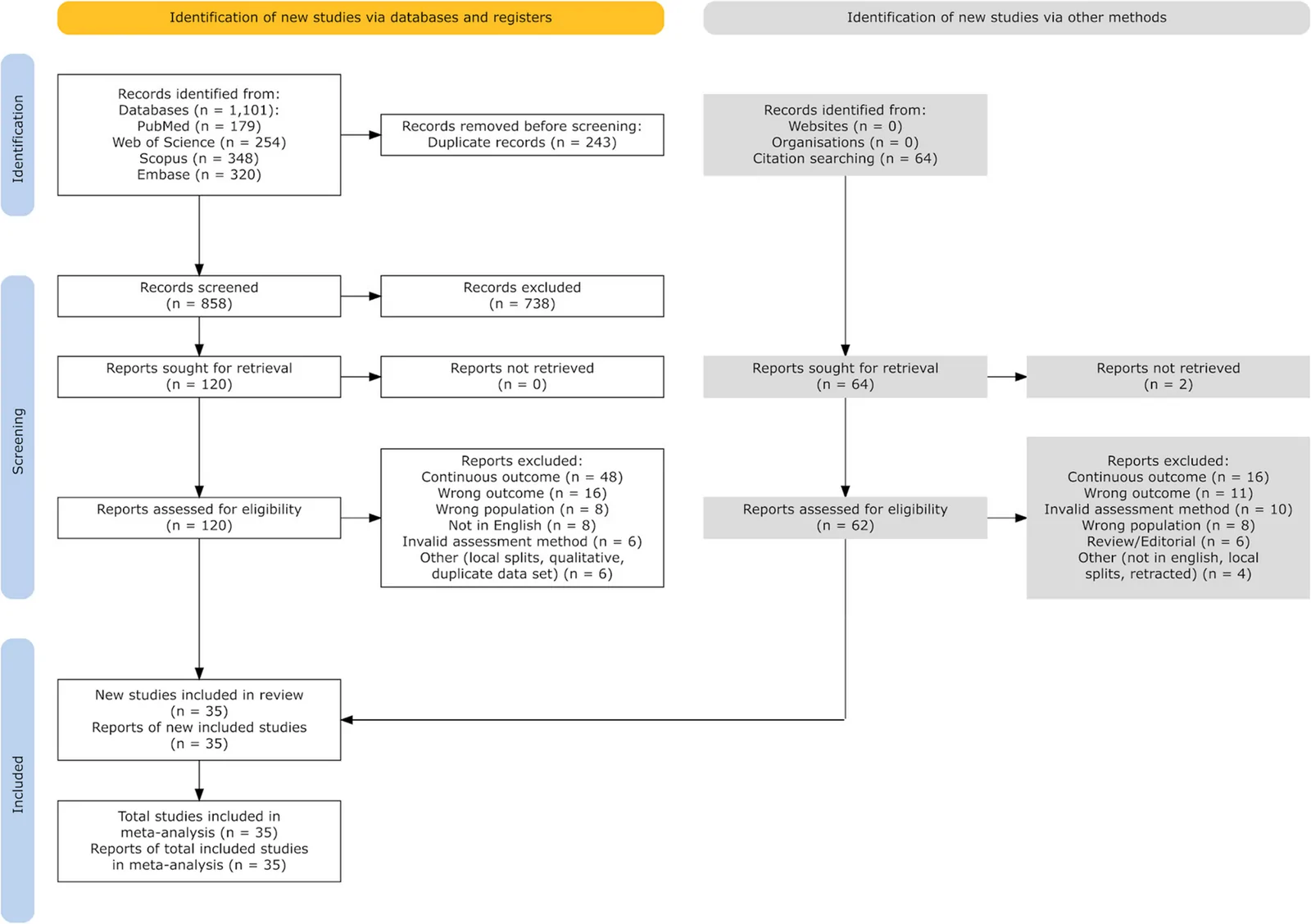
PRISMA 2020 flow diagram of study identification and selection

Backward and forward citation searching identified 64 additional potentially relevant studies. Of these, 62 were retrieved and assessed for eligibility, of which 7 studies met the inclusion criteria and were included in the review, for a total of 35 included studies. Figure 1 presents the PRISMA flow diagram detailing the study identification and selection process.

### Study characteristics

The characteristics of the included studies are presented in Table 1. A total of 35 studies, comprising 13,489 medical students, met the criteria for inclusion in the systematic review. The pooled percentage of female participants was approximately 61.8%, and the mean age of participants clustered around 21 years (Table 1).

**Table 1:** Summary of studies included in the systematic review and meta-analysis

Study ID	Country	Study Design	Administration timing	Tool used	Medical students’ characteristics^1^	TA prevalence (%)	Key findings^1^
Bharti et al., 2026 [36]	India	Interventional	Pre-examination	WTAS	100 students, 56% female, NR mean age	40	A peer-led mentoring program significantly reduced test anxiety scores and completely eliminated severe anxiety, though academic performance did not significantly improve.
Bischofsberger et al., 2021 [13]	Germany	Repeated Measures Cross-Sectional	Pre-examination	Prüfungsangstfragebogen (PAF)	625 students, 55.8% female, 21.2 (3.9) mean age	10.0	Worry showed the highest prevalence rates (up to 48%), while interference showed the lowest prevalence rates (approximately 5%).
Darwish et al., 2026 [37]	Egypt	Cross-sectional	Pre-, during, and post-examinations	WTAS	2437 students, 56.5% female, 20.0 (1.9) mean age	46.9	Lower odds of test anxiety were associated with completing the questionnaire during or after exams, greater satisfaction with academic performance, attending college once or twice weekly, participating in extracurricular activities, and sleeping more than six hours per night. Higher odds were associated with group studying, enrolling in private courses for all subjects, and increasing levels of psychological distress.
Green et al., 2016 [1]	USA	Cross-sectional	Pre-examination	WTAS	93 students, 43% female, NR mean age	25.8	Test anxiety was modestly inversely correlated with USMLE step-1 scores. A test-taking strategies course modestly reduced test anxiety among cases, while anxiety in controls increased.
Mastour et al., 2022 [38]	Iran	Cross-sectional	Pre-examination	TAS	290 students, 66.9% female, NR mean age	72.1	Female students’ test anxiety was significantly higher than that of male students, and participants over 20 years old had significantly higher test anxiety scores.
Memon et al., 2023 [39]	Saudi Arabia	Cross-sectional	Pre-examination	WTAS	209 students, 34% female, NR mean age	47.4	Test anxiety was higher among females compared to males. Using lectures alone or with lecture recordings was associated with lower test anxiety compared to using other resources.
Mosavi et al., 2023 [40]	Iran	Interventional	Pre-examination	TAS	114 students, 49.1% female, 21.9 (3.1) mean age	70.7	Shen Men auricular acupressure significantly reduced mean test anxiety and improved anxiety intensity compared to controls.
Patel et al., 2023 [41]	India	Cross-sectional	Pre-examination	WTAS	369 students, 60.4% female, 20.4 (1.34) mean age	60.2	No significant association between test anxiety and gender.
Sohail et al., 2020 [42]	Pakistan	Cross-sectional	Pre-examination	WTAS	409 students, 72% female, NR mean age	72	Poor academic performance was associated with high to extremely high pre-exam anxiety while high achievers had relatively lower anxiety levels.
Saravanan and Kingston, 2014 [43]	Malaysia	Interventional	Pre-examination	WTAS	436 students, NR female, 19 (1.04) mean age	17.9	Following the intervention, the intervention group showed reduced anxiety, psychological distress, and amotivation, and increased intrinsic and extrinsic motivation at post-assessment compared with pre-assessment (all *p* < 0.01).
Shaheen et al., 2022 [44]	Pakistan	Cross-sectional	Pre-examination	TAQ	82 students, 57.7% female, 21.26 (1.23) mean age	23.2	Mean test anxiety levels differed significantly across assessment stages (*p* < 0.001), and anxiety was significantly negatively correlated with academic scores among female students.
Simran et al., 2015 [12]	India	Repeated Measures Cross-Sectional	Pre-examination	VAS	110 students, 58.2% female, NR mean age	99.1	Female students had higher test anxiety compared to male students.
Tata et al., 2024 [45]	India	Cross-sectional	Pre-examination	WTAS	106 students, 60.4% female, NR mean age	41.5	Female students had higher test anxiety compared to male students.
Alshiblawy et al., 2024 [46]	Iraq	Cross-sectional	During examinations	TAQ	579 students, 66.3% female, NR mean age	31.3	Females reported significantly higher anxiety levels than males, and younger students (18–21 years old) exhibited higher anxiety.
Aziz and Serafi − 2017 [47]	Saudi Arabia	Cross-sectional	During examinations	WTAS	411 students, 100% female, 21.7 (NR) mean age	53	A positive linear correlation between test anxiety and psychological distress was seen with advancing years of the course.
Jirjees et al., 2024 [48]	UAE	Cross-sectional	During examinations	WTAS	347 students, NR female, NR mean age	63.4	Test anxiety was moderately high and most prevalent among females, younger students, junior students, and those with low cGPAs.
Khan et al., 2013 [49]	Pakistan	Cross-sectional	During examinations	VAS	200 students, 38.5% female, 19.7 (0.8) mean age	64.0	Prevalence of exam anxiety was higher in 1st year students (73.46%) compared to 2nd year students (54.90%). Most important factors contributing to exam anxiety were inadequate rest (89%), irrational thoughts (67.50%), and excessive course load (60%).
Liu et al., 2021 [15]	China	Cross-sectional	During examinations	TAS	1266 students, 64.8% female, NR mean age	71.4	Male college students exhibited lower test anxiety compared to female college students, and students with excellent academic performance had lower test anxiety compared to those with poor academic evaluation.
Sindhu et al., 2023 [50]	India	Cross-sectional	During examinations	WTAS	172 students, 60.5% female, NR mean age	56.4	A negative correlation was seen between pre university course marks and test anxiety scores
Ali et al., 2015 [51]	Pakistan	Cross-sectional	Post-examination	WTAS	387 students, 77.8% female, 19.7 (1.0) mean age	35.9	Students in the pass/fail assessment system had a significantly lower test anxiety score compared to students in the GPA system.
Patil and Aithala, 2017 [14]	India	Cross-sectional	Post-examination	WTAS	300 students, 50% female, 18–23 (range) mean age	33.3	Test anxiety was significantly higher among males.
Roy et al., 2022 [52]	India	Cross-sectional	Post-examination	WTAS	365 students, 35.6% female, NR mean age	56.2	Male gender, addiction to smoking and alcohol, virtual gaming, and residing at home during exams were associated with higher levels of test anxiety, while regular yoga practice was identified as a protective factor.
Afzal et al., 2012 [53]	Pakistan	Cross-sectional	Not specified	WTAS	388 students, 74% female, 20.82 (1.17) mean age	33.5	Female medical students showed higher levels of anxiety compared to male students, and students in their fourth and final years of studies showed a higher level of anxiety compared to those in the 2nd and 3rd years.
Almutairi et al., 2024 [16]	Saudi Arabia	Cross-sectional	Not specified	WTAS	205 students, 57.6% female, NR mean age	33.7	Students with poor social support exhibited increased odds of high anxiety. Perception of increased anxiety since the previous year was a significant predictor of high anxiety.
Arain − 2021 [54]	Saudi Arabia	Cross-sectional	Not specified	TAQ	518 students, 38.42% female, 23 (1.3) mean age	17.8	Anxiety had a negative effect on overall academic performance.
Ćurčić et al., 2024 [55]	Bosnia and Herzegovina	Cross-sectional	Not specified	WTAS	145 students, 69.7% female, NR mean age	59.3	Elevated levels of test anxiety were positively correlated with increased consumption of psychoactive compounds.
Farnia et al., 2017 [56]	Iran	Cross-sectional	Not specified	TAS	196 students, 28.6% female, 22.39 (2.08) mean age	82.7	Students utilizing problem-focused coping strategies demonstrated higher emotional intelligence and lower test anxiety; emotional intelligence was found to be a key mediator that significantly reduces test anxiety as it increases.
Lin et al., 2025 [57]	China	Cross-sectional	Not specified	TAS	420 students, 51.9% female, NR mean age	54.7	Academic self-efficacy and brief fear of negative evaluation indirectly mediate the relationship between neurotic personality traits and test anxiety.
Rehman et al., 2018 [58]	Pakistan	Cross-sectional	Not specified	VAS	300 students, 55% female, 22.14 (2.44) mean age	88.7	Younger students had significantly higher test anxiety than older students.
Rtbey et al., 2022 [18]	Ethiopia	Cross-sectional	Not specified	WTAS	420 students, 39.5% female, 22.86 (1.85) mean age	29.0	High test anxiety was a significant predictor of mental distress.
Sadaf et al., 2022 [59]	Pakistan	Cross-sectional	Not specified	TAQ	250 students, 100% female, 20.1 (0.6) mean age	27.2	Most (77%) experienced moderate anxiety.
Sharma et al., 2023 [60]	India	Cross-sectional	Not specified	WTAS	230 students, 50.4% female, 21.3 (1.9) mean age	49.6	Excessive course load, psychological stress, and low self-esteem were identified as independent risk factors for test anxiety.
Song et al., 2024 [61]	China	Interventional	Not specified	TAS	250 students, NR% female, NR mean age	25.6	Students received group impromptu music therapy reported significantly lower levels of test anxiety, experienced fewer difficulties with emotional regulation, and achieved higher examination scores.
Tsegay et al., 2019 [17]	Ethiopia	Cross-sectional	Not specified	WTAS	390 students, 40.5% female, 21.8 (1.72) mean age	52.3	Female sex, lower academic grades, first-year status, excessive course load, and oral examinations were identified as predictors of test anxiety among medical students.
Zhang et al., 2022 [19]	China	Interventional	Not specified	TAS	598 students, NR female, NR mean age	17.1	Hypnosis was more effective than PMR in reducing test anxiety among medical students.

*Abbreviations: TA *Test Anxiety, *TAQ *Test Anxiety Questionnaire, *TAS *Test Anxiety Scale, *WTAS *Westside Test Anxiety Scale, *VAS *Visual Analogue Scale, *PAF *Prüfungsangstfragebogen, *PMR *Progressive Muscle Relaxation, *cGPA *cumulative Grade Point Average, *USMLE *United States Medical Licensing Examination, *NR *Not Reported, *NA *Not Available, *UAE *United Arab Emirates, *USA *United States of America

^1^Total sample size (*n*), percentage of female participants (%), and mean age with standard deviation (SD), unless otherwise specified

Of the 35 included studies, the vast majority utilized a cross-sectional design (28 studies). An additional five were interventional studies [19, 36, 40, 43, 61], and two utilized repeated measures cross-sectional designs [12, 13]. The studies were published between 2012 and 2026, and were conducted across 12 countries. The highest research output originated from India (8 studies) and Pakistan (7 studies), followed by Saudi Arabia and China (4 studies each).

Regarding the instruments utilized, the WTAS was the most frequently administered tool (20 studies). Other instruments included the TAS (7 studies), the TAQ (4 studies), and the VAS (3 studies). Only one study utilized the German-language Prüfungsangstfragebogen (PAF). The cutoffs used in each of the studies are summarized in Table S2 (Supplementary Material 1).

Overall, the risk of bias across the 35 included studies was variable. The majority of the studies (*n* = 28, 80.0%) represented their target population. Specifically, 10 studies (28.6%) were truly representative (utilizing random sampling or including all subjects), and 18 studies (51.4%) were somewhat representative (utilizing non-random sampling). The remaining 7 studies (20.0%) failed to provide a description of their sampling strategy. However, only a minority of the studies (*n* = 13, 37.1%) calculated the required sample size. Almost all studies (*n* = 32, 91.4%) utilized an acceptable assessment tool to evaluate the primary outcome, while 3 studies (8.6%) relied on non-acceptable assessment methods (i.e. the VAS). See Table S3 (Supplementary Material 1) for a detailed risk of bias assessment.

### Overall prevalence of test anxiety

**Fig. 2:**
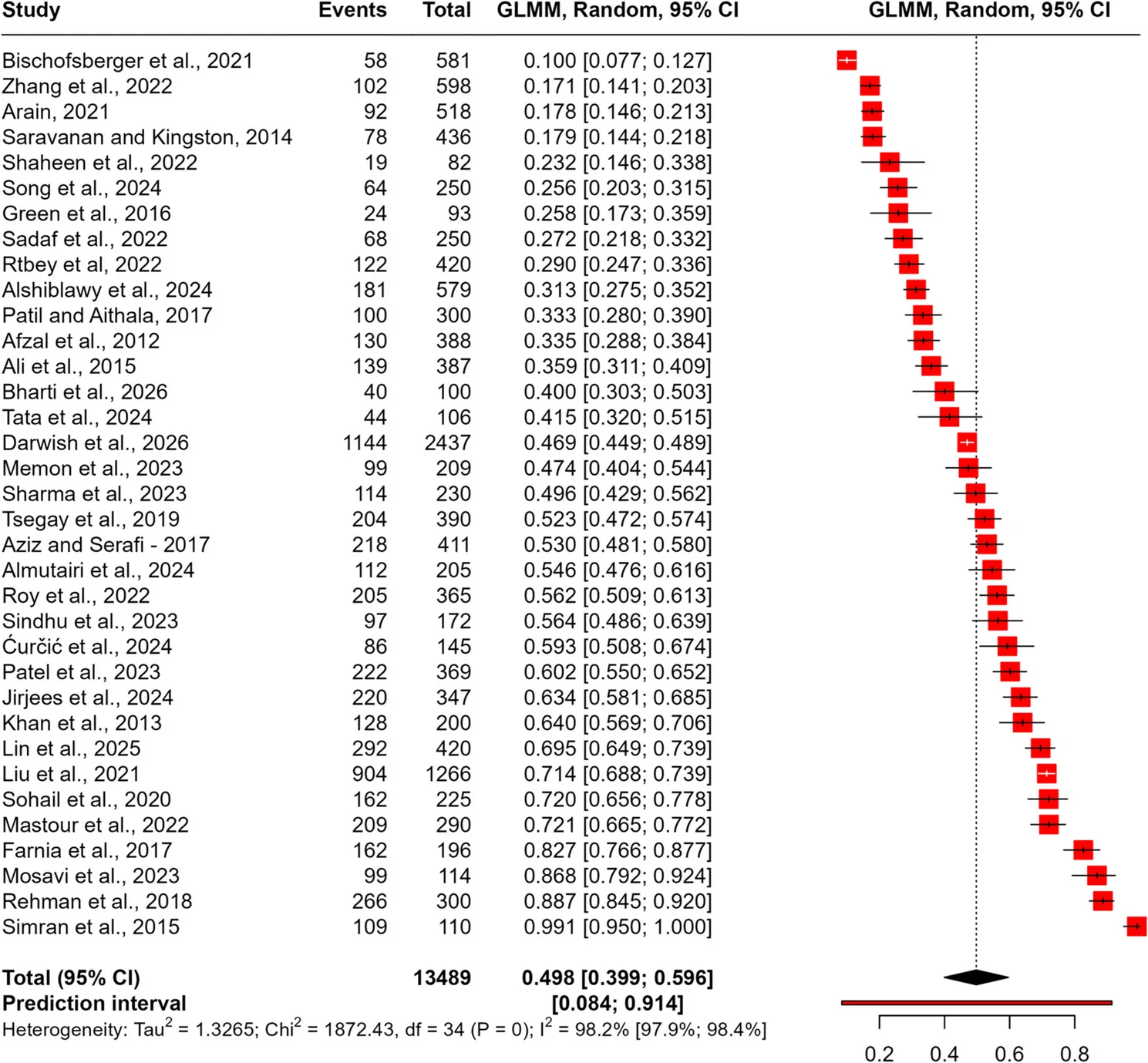
Forest plot of the prevalence of test anxiety among medical students

The meta-analysis included 35 studies reporting on the prevalence of test anxiety among medical students, comprising a total of 13,489 participants and 6,313 events. Using a random-effects model, the overall pooled prevalence of test anxiety was 49.8% (95% CI: 39.9%, 59.6%). A high degree of absolute heterogeneity was observed across the included studies (Tau² = 1.33). The 95% prediction interval ranged from 8.4% to 91.4%, indicating substantial variability in true prevalence across different settings (Fig. 2).

### Subgroup analyses

**Fig. 3:**
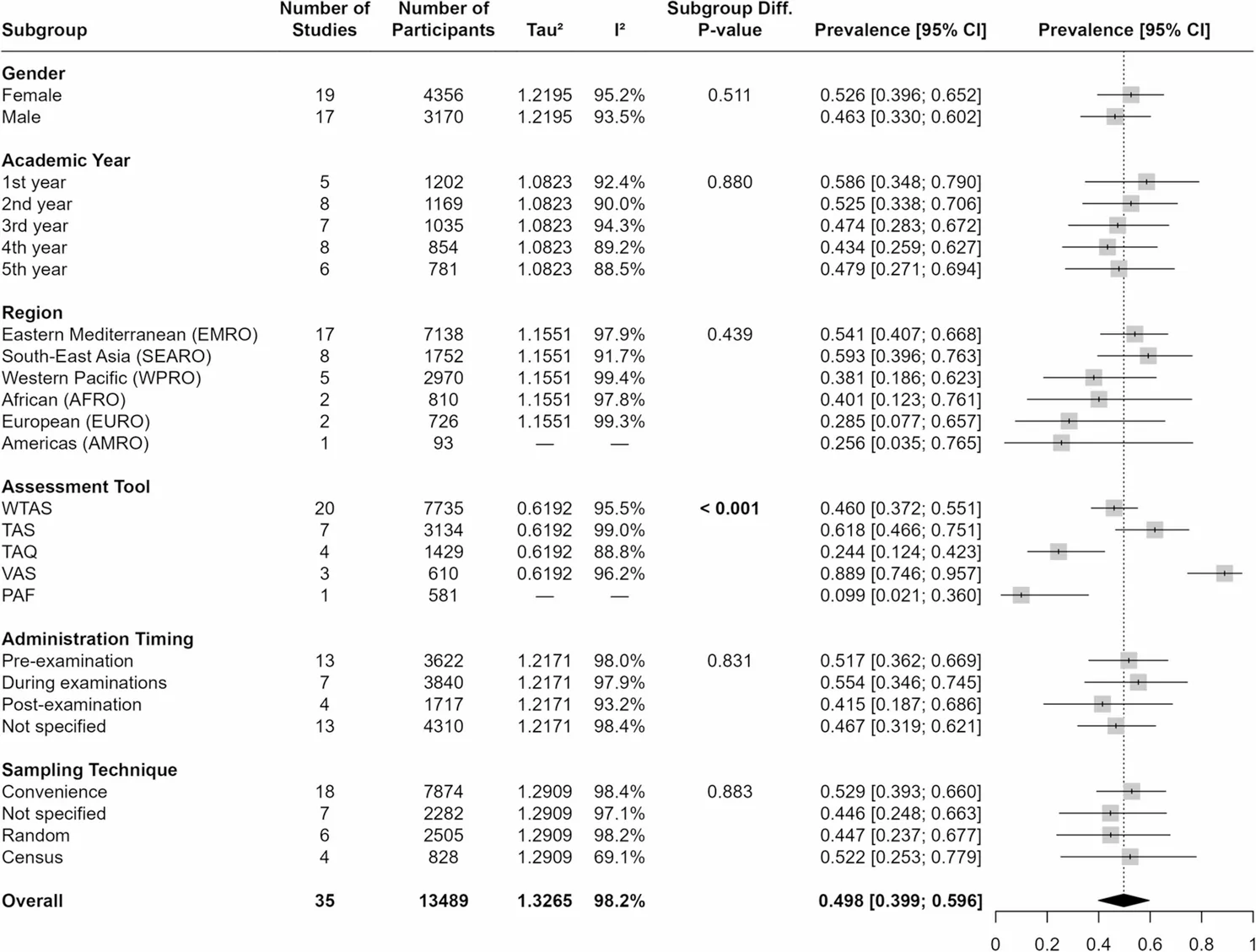
Summary forest plot of subgroup analyses for the prevalence of test anxiety among medical students

Subgroup analyses were conducted based on gender, academic year, region, assessment tool, administration timing, and sampling technique (summarized in Fig. 3).

Gender was not significantly associated with differences in the prevalence of test anxiety (*p* = 0.511), with a residual Tau² of 1.22. The pooled prevalence was 52.6% (95% CI: 39.6%, 65.2%) among female medical students across 19 studies and 46.3% (95% CI: 33.1%, 60.2%) among male medical students across 17 studies (Figure S1; Supplementary Material 1).

Prevalence was highest among first year students (58.6%, 95% CI: 34.8%, 79.0%) and second year students (52.6%, 95% CI: 33.8%, 70.6%), and appeared slightly lower in subsequent years (47.4% in the third year, 43.4% in the fourth year, and 47.9% in the fifth year). However, differences across academic years were not statistically significant (*p* = 0.880), with a residual Tau² of 1.08 (Figure S2; Supplementary Material 1).

Regional differences were not statistically significant (*p* = 0.439), with a residual Tau² of 1.16. Studies from the South-East Asia region (SEARO) reported the highest prevalence at 59.3% (95% CI: 39.6%, 76.3%), while the Eastern Mediterranean region (EMRO), which contributed the largest number of studies (17), had a prevalence of 54.1% (95% CI: 40.7%, 66.8%). Regions with fewer studies showed varied estimates (Figure S3; Supplementary Material 1).

The choice of assessment tool was significantly associated with differences in the reported prevalence of test anxiety (*p* < 0.001). Notably, subgrouping by assessment tool resulted in the largest decrease in heterogeneity, lowering the residual Tau² to 0.62. Studies using the Visual Analogue Scale (VAS) reported the highest prevalence (88.9%, 95% CI: 74.6%, 95.7%), while those using the Test Anxiety Questionnaire (TAQ) reported the lowest estimates (24.4%, 95% CI: 12.4%, 42.3%). The Westside Test Anxiety Scale (WTAS), which was the most commonly used tool (20 studies), yielded a prevalence of 46.0% (95% CI: 37.2%, 55.1%) (Figure S4; Supplementary Material 1).

Administration timing did not yield statistically significant differences (*p* = 0.831), with a residual Tau² of 1.22. Estimates were highest when assessed during examinations (55.4%, 95% CI: 34.6%, 74.5%) compared to both pre-examination (51.7%) and post-examination (41.5%) periods (Figure S5; Supplementary Material 1).

Sampling technique was not significantly associated with differences in the prevalence of test anxiety (*p* = 0.883), with a residual Tau² of 1.29. Studies using convenience sampling reported the highest prevalence (52.9%, 95% CI: 39.3%, 66.0%) across 18 studies, compared to those using census sampling (52.2%), random sampling (44.7%), and unspecified sampling (44.6%) (Figure S6; Supplementary Material 1).

### Meta-regression

**Fig. 4:**
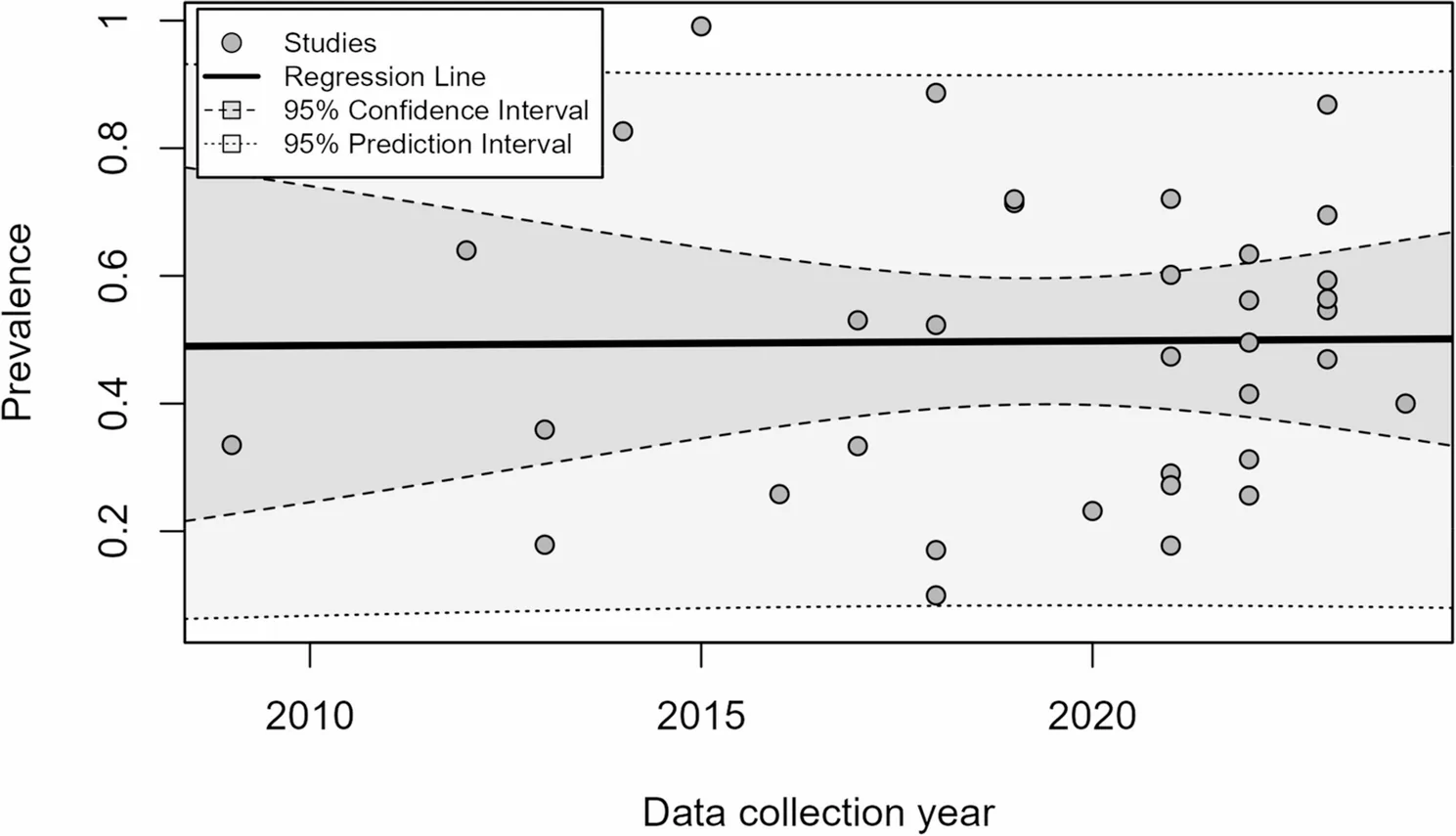
Bubble plot of meta-regression analysis showing the association between data collection year and the prevalence of test anxiety among medical students

A meta-regression was conducted to examine the effect of data collection year on the prevalence of test anxiety. The results indicated that data collection year was not significantly associated with the reported prevalence of test anxiety (coefficient = 0.003, 95% CI: -0.105, 0.111, *p* > 0.9), with a residual Tau² of 1.33 (Table S4; Supplementary Material 1). This lack of association is illustrated in the bubble plot (Fig. 4).

### Sensitivity analysis

A leave-one-out sensitivity analysis was performed to determine if any single study heavily influenced the overall pooled prevalence. The omission of any individual study yielded pooled prevalence estimates ranging between 47.1% and 51.3%, indicating that the overall estimate was relatively consistent. However, the omission of the study by Simran et al., which reported an extreme prevalence of 99.1% using the non-validated VAS, resulted in the largest decrease in heterogeneity, reducing Tau² to 0.97 compared to the overall Tau² of 1.33 (Figure S7; Supplementary Material 1).

To assess the impact of studies utilizing the VAS on the overall effect sizes, an additional sensitivity analysis was conducted excluding these studies. The exclusion of VAS studies (k = 32, *n* = 12,879) resulted in a lower pooled prevalence of 44.9% (95% CI: 36.7%, 53.4%), alongside a notable decrease in heterogeneity (Tau² = 0.86) compared to the main analysis. Subgroup analyses and meta-regression excluding VAS-based studies yielded results consistent with the primary findings; detailed outputs are provided in Supplementary Material 1 (Figures S8–S18 and Table S5).

**Fig. 5:**
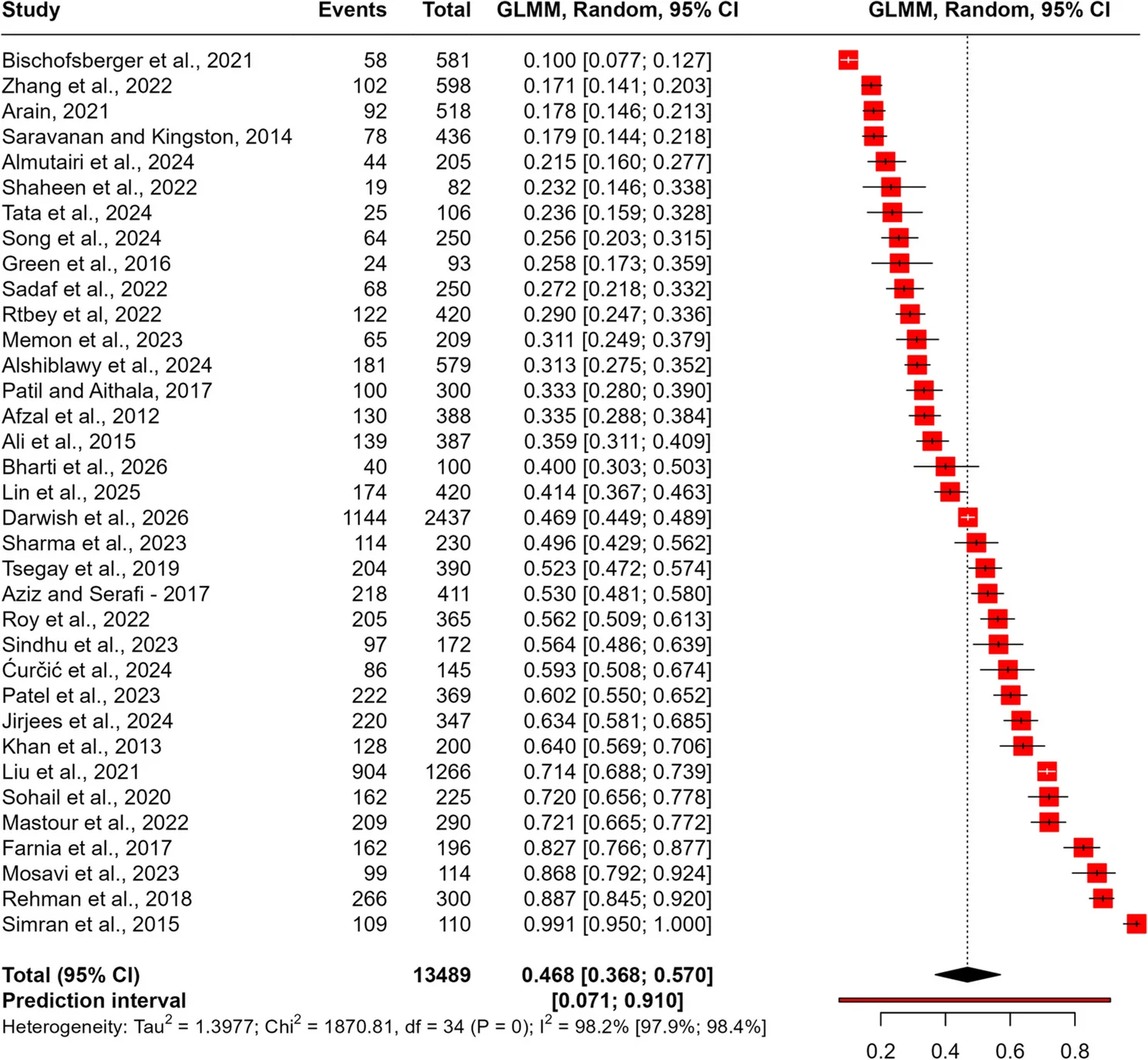
Forest plot of the prevalence of test anxiety among medical students in the sensitivity analysis including only severe/high test anxiety categories from studies that combined low and moderate anxiety levels into a single category

To assess the impact of studies that combined low and moderate test anxiety levels into a single category [16, 39, 45, 57], an additional sensitivity analysis was performed. While the main analysis imputed their overall test anxiety by halving this combined number and adding it to the severe category, this sensitivity analysis included strictly their severe/high test anxiety categories exclusively, yielding a pooled prevalence of 46.8% (95% CI: 36.8%, 57.0%) (Fig. 5).

## Discussion

The present systematic review and meta-analysis pooled data from 35 studies comprising 13,489 medical students across multiple world regions, yielding a pooled prevalence of 49.8%, which decreased to 44.9% upon exclusion of VAS-based studies. This estimate positions test anxiety as affecting nearly one in two medical students globally, a figure that warrants attention from medical educators and institutional policymakers alike. Nevertheless, this finding should be interpreted with caution due to the substantial heterogeneity observed, largely driven by variability in the measurement tools used across the included studies.

Our estimate is nearly identical to the 48.4% reported by Ansari and Iqbal in their meta-analysis examining exam anxiety across mixed student populations [25], and to their healthcare-specific subgroup estimate of 47.3%. However, our estimate is considerably higher than the 33.8% prevalence of general anxiety among medical students reported by Quek et al. [62], a finding consistent with the expectation that examination-specific distress should exceed background trait-level anxiety in this population.

Importantly, we observed substantial heterogeneity across included studies, a finding consistent with prior meta-analyses pooling cross-sectional estimates of psychiatric symptom prevalence among medical students [25, 62, 63], and one that cautions against interpreting the pooled estimate in isolation. Subgroup analysis by assessment tool was the most informative source of heterogeneity, yielding a statistically significant between-group difference. Studies using the TAS reported the highest prevalence among validated instruments (61.8%), followed by the WTAS (46%) and TAQ (24%), while VAS-based studies produced a markedly elevated estimate of 89%, including one study in which nearly all participants met the threshold for test anxiety (99%) [12]. This pattern is likely attributable to the limitations of the VAS as a test-anxiety measure: unlike validated instruments such as the WTAS, TAQ, and TAS, the VAS lacks psychometric validation in this context. To examine the influence of instrument choice on the pooled estimate, we conducted a sensitivity analysis excluding VAS studies, which reduced the pooled prevalence to 44.9%, a finding that highlights how substantially reported prevalence is sensitive to instrument selection. Accordingly, our 49.8% primary estimate, and the 44.9% estimate obtained after excluding VAS-based studies, should be viewed as bracketing the plausible range of true prevalence depending on the strictness of the operational definition.

This measurement heterogeneity is further compounded by the lack of consensus on severity and case classification. A systematic review of leveling strategies in test-anxiety research found that most studies either treat test anxiety as a continuous variable without defining caseness or rely on methodologically weak approaches such as single-sample splits [22]. As a result, cross-study prevalence comparisons remain difficult even when the same instrument is used. In an attempt to mitigate this, we harmonized severity classifications across instruments by defining a case as any report within the moderate–high to severe/extreme range, applied conservative rules when categories were merged, and excluded studies relying on single-sample splits. By comparison, Ansari and Iqbal [25], applied a broader case definition, classifying students with ‘high-normal’ scores on the WTAS as cases and including measures not specific to test anxiety such as the Depression, Anxiety, and Stress Scale (DASS).

Beyond measurement instrument considerations, our findings also highlight the impact of administration timing. Although subgroup analysis by timing of questionnaire administration did not yield statistically significant differences, the observed pattern offers relevant insight when interpreted cautiously. Estimated prevalence was highest during examination periods (55.4%), followed by pre-examination (51.7%), and lowest post-examination (41.5%). This aligns with the transactional process model of test anxiety, which posits that state anxiety intensifies during the anticipatory phase and peaks immediately prior to assessment [9]. However, because test anxiety is a temporally sensitive and context-dependent construct, the pooled prevalence estimate should be interpreted with caution. Notably, 13 out of the 35 included studies did not explicitly report administration timing relative to examinations, which may have introduced misclassification and attenuated true between-group differences. Furthermore, the “pre-examination” category encompassed a broad and heterogeneous time frame, ranging from days to several weeks or months before an exam. This variability likely attenuated the true pre-exam peak, rendering the “during examination” category a more accurate proxy for maximal evaluative threat. Future studies should therefore report administration timing with greater precision — specifying the exact interval between questionnaire completion and the relevant examination — to enable more meaningful comparisons across studies and strengthen the evidence base for temporally sensitive prevalence estimates.

In our study, subgroup analysis by gender revealed that female medical students reported numerically higher test anxiety than their male counterparts (52.6% vs. 46% respectively), yet this difference did not reach statistical significance in either analysis. This null finding is consistent with Quek et al. [62] and Lin et al. [64], who similarly found no significant gender difference in general anxiety among medical students, and is similar to the findings of Ansari and Iqbal [25], whose subgroup estimates for females (54.96%) were higher than males (46.0%), although they didn’t conduct subgroup differences analysis. Taken together, these findings suggest that the well-documented higher test anxiety prevalence among females [9] may attenuate in medical student populations specifically. This is consistent with the findings of von der Embse et al. in their 30-year review of test anxiety across educational levels [4], who found that while females report significantly higher test anxiety than males overall, this gender discrepancy decreases at the postsecondary level. One explanation is that medical school admission selects for high-achieving, performance-oriented individuals regardless of gender, compressing the distribution of test anxiety in both groups and reducing the between-gender variance that is more apparent in general student populations. Institutional interventions targeting test anxiety in medical students should therefore be designed with both genders in mind rather than assuming disproportionate female vulnerability.

We found that test anxiety was highest in first year students (58.6%), and decreased by nearly 10–15% among fourth and fifth year students. Nevertheless, subgroup analysis by academic year showed no statistically significant differences. This finding is consistent with previous meta-analytic evidence reporting no significant differences in anxiety levels between pre-clinical and clinical medical students [62, 64]. Future longitudinal studies are needed to better clarify how test anxiety may change across different stages of medical training.

Subgroup analysis by world region showed the highest prevalence in SEARO (59%), followed closely by EMRO (54%), with no statistically significant differences in test anxiety prevalence between regions. However, this finding should be interpreted cautiously, as non-English studies were excluded, estimates for Europe and Africa were each based on two studies, and the South-East Asia estimate decreased significantly after excluding VAS-based studies. Even so, the absence of significant regional differences contrasts with Quek et al., who reported notable geographic variation in general anxiety among medical students [62], with the Middle East showing the highest prevalence (42.4%), followed by Asia (35.2%). Similarly, Ansari and Iqbal [25] in their mixed-population analysis of student exam anxiety ranked the Middle East as the highest-prevalence region for exam anxiety. The elevated prevalence observed in South-East Asia may be understood in light of Stankov’s argument that educational cultures in Asia emphasizing effort over innate ability, coupled with stringent academic expectations, can foster heightened anxiety [65]. Nonetheless, future work should expand geographic coverage, especially in Europe, America, and Africa.

Lastly, our meta-regression did not identify a statistically significant association between publication year and the prevalence of test anxiety among medical students. In contrast, Oswalt et al., in a secondary analysis of the American College Health Association–National College Health Assessment II (2009–2015), reported a progressive rise in anxiety diagnoses and treatment among college students [66]. Similarly, Lin et al. observed a positive correlation between survey year and anxiety prevalence among medical students [64], and Li et al. reported significantly higher anxiety rates following the COVID-19 pandemic [67]. This discrepancy may be explained by the differences in constructs, as unlike general anxiety, test anxiety is context-bound and may be less sensitive to broader temporal trends driven by sociocultural or healthcare-related changes.

Given the high prevalence of test anxiety identified in this review, medical schools should move beyond simply recognizing test anxiety and implement structured support systems. Routine screening for test anxiety may be particularly valuable before examinations. Early identification can facilitate timely interventions, including cognitive-behavioral techniques, mindfulness-based approaches, and study skills training. Institutions should also prioritize the use of validated assessment tools, such as the WTAS or TAS, rather than non-validated measures like the VAS, to ensure accurate identification of affected students and reliable evaluation of support programs over time.

### Limitations

This systematic review and meta-analysis has several limitations. Substantial heterogeneity was observed across studies, reflecting variability in study populations, assessment tools, severity thresholds, timing of administration, and institutional contexts. Although subgroup and sensitivity analyses explored potential sources of heterogeneity, much of it remained unexplained, limiting the interpretability and generalizability of pooled prevalence estimates. Most included studies relied on self-reported, cross-sectional survey data, which are susceptible to recall and social desirability biases and preclude causal or longitudinal inferences. The geographic distribution of studies was uneven, with a predominance of research from SEARO and the EMRO and limited representation from Africa, Latin America, and other underrepresented regions, constraining global generalizability. Instrument choice and inconsistent operationalization of severity further complicate comparisons across studies. The use of validated instruments (WTAS, TAS, TAQ, VAS), alongside the non-validated VAS, and variable cutoffs likely influenced reported prevalence and contributed to residual heterogeneity. Differences in risk of bias, including the inclusion of unsatisfactory-quality studies—particularly all VAS-based studies—may have inflated prevalence estimates. Finally, the reliance on published literature and exclusion of non-English studies may have introduced selection bias, potentially omitting relevant data and affecting the representativeness of findings.

Search limitations may also have affected completeness of the evidence base. We did not search PsycINFO, and authors of studies reporting only continuous test anxiety scores were not contacted for additional data, which may have reduced the number of eligible studies and affected the precision of the pooled estimate. In addition, restriction to published, English-language literature may have introduced selection bias.

Finally, certainty of evidence was not formally assessed due to the absence of a validated tool specifically tailored for prevalence meta-analyses in this context; therefore, all findings should be interpreted with appropriate caution.

## Conclusions

Test anxiety is highly prevalent among medical students, affecting nearly half of the population. Estimates are influenced by assessment tools, severity definitions, and timing of measurement, highlighting substantial heterogeneity across studies. These findings underscore the need for standardised instruments, validated cutoffs, and careful reporting of administration timing in future research. Routine, evidence-based interventions to identify and support students at risk are essential to mitigate the academic and psychological impact of test anxiety.

## Supplementary Information

Supplementary Material 1.

## Data Availability

The dataset supporting the findings of this study are available from the corresponding author upon reasonable request.

## References

[1] Green M, Angoff N, Encandela J. Test anxiety and United States medical licensing examination scores. Clin Teach. 2016;13:142–6. 10.1111/tct.12386. 26037042

[2] Sarason IG, Sarason BR. Test Anxiety. In: Leitenberg H, editor. Handbook of Social and Evaluation Anxiety. Boston, MA: Springer US; 1990. pp. 475–95. 10.1007/978-1-4899-2504-6_16.

[3] Endler NS, Kocovski NL. State and trait anxiety revisited. J Anxiety Disord. 2001;15:231–45. 10.1016/S0887-6185(01)00060-3. 11442141

[4] Von Der Embse N, Jester D, Roy D, Post J. Test anxiety effects, predictors, and correlates: a 30-year meta-analytic review. J Affect Disord. 2018;227:483–93. 10.1016/j.jad.2017.11.048. 29156362

[5] Thames AD, Panos SE, Arentoft A, Byrd DA, Hinkin CH, Arbid N. Mild test anxiety influences neurocognitive performance among African Americans and European Americans: identifying interfering and facilitating sources. Cultur Divers Ethnic Minor Psychol. 2015;21:105–13. 10.1037/a0037530. 25111554 PMC5567740

[6] Keeley J, Zayac R, Correia C. Curvilinear relationships between statistics anxiety and performance among undergraduate. students: evidence for optimal anxiety. SERJ. 2008;7:4–15. 10.52041/serj.v7i1.477.

[7] Eysenck MW. Anxiety, learning, and memory: a reconceptualization. J Res Pers. 1979;13:363–85. 10.1016/0092-6566(79)90001-1.

[8] Wine JD. Evaluation anxiety: a cognitive-attentional construct. In: Krohne HW, Laux L, editors. Achievement, stress, and anxiety. Washington, DC: Hemisphere; 1982. pp. 207–19.

[9] Zeidner M. Test anxiety: the state of the art. Springer Science & Business Media. 1998.

[10] Wadi MM, Yusoff MSB, Rahim AFA, Lah NAZN. Factors influencing test anxiety in health professions education students: a scoping review. SN Soc Sci. 2022;2:174. 10.1007/s43545-022-00459-9.

[11] Schaefer A, Mattheß H, Pfitzer G, Köhle K. Seelische Gesundheit und Studienerfolg von Studierenden der Medizin mit hoher und niedriger Prüfungsängstlichkeit. Psychother Psych Med. 2007;57:289–97. 10.1055/s-2006-951974. 17357900

[12] Grewal S, Nagpal S, Walia L. Evaluation of examination anxiety status and its associated factors among first professional medical (MBBS) students. Int J Interdisciplinary Multidisciplinary Stud. 2015;2(8):1–11.

[13] Bischofsberger L, Burger PHM, Hammer A, Paulsen F, Scholz M, Hammer CM. Prevalence and characteristics of test anxiety in first year anatomy students. Annals Anat - Anatomischer Anzeiger. 2021;236:151719. 10.1016/j.aanat.2021.151719. 33677044

[14] Patil SG, Aithala MR. Exam anxiety: its prevalence and causative factors among Indian medical students. Natl J Physiol Pharm Pharmacol. 2017;7(12):1323–8. 10.5455/njppp.2017.7.0516113072017.

[15] Liu Y, Pan H, Yang R, Wang X, Rao J, Zhang X, et al. The relationship between test anxiety and emotion regulation: the mediating effect of psychological resilience. Ann Gen Psychiatry. 2021;20:40. 10.1186/s12991-021-00360-4. 34488816 PMC8419945

[16] Almutairi AG, Baabbad NM, Alhumaidan AA, Alshahrani AM, Alabdulkarim AI, Alsughier N. Prevalence and factors causing test anxiety among medical students. Middle East Curr Psychiatry. 2024;31:48. 10.1186/s43045-024-00437-2.

[17] Tsegay L, Shumet S, Damene W, Gebreegziabhier G, Ayano G. Prevalence and determinants of test anxiety among medical students in Addis Ababa Ethiopia. BMC Med Educ. 2019;19:423. 10.1186/s12909-019-1859-5. 31727023 PMC6857229

[18] Rtbey G, Shumet S, Birhan B, Salelew E. Prevalence of mental distress and associated factors among medical students of University of Gondar, Northwest Ethiopia: a cross-sectional study. BMC Psychiatry. 2022;22:523. 10.1186/s12888-022-04174-w. 35918673 PMC9345005

[19] Zhang Y, Yang X-X, Luo J-Y, Liang M, Li N, Tao Q, et al. Randomized trial estimating effects of hypnosis versus progressive muscle relaxation on medical students’ test anxiety and attentional bias. WJP. 2022;12:801–13. 10.5498/wjp.v12.i6.801. 35978973 PMC9258271

[20] Khoshhal KI, Khairy GA, Guraya SY, Guraya SS. Exam anxiety in the undergraduate medical students of Taibah University. Med Teach. 2017;39:S22–6. 10.1080/0142159X.2016.1254749. 28103727

[21] Reteguiz J-A. Relationship between anxiety and standardized patient test performance in the medicine clerkship. J Gen Intern Med. 2006;21:415–8. 10.1111/j.1525-1497.2006.00419.x. 16704380 PMC1484796

[22] Cassady JC, Tan SH, Robiullah A, Badzovski I, Janiuk J. Methods employed in studies identifying levels of test anxiety in university students: a systematic review. Behav Sci. 2025;15(3):331. 10.3390/bs15030331. 40150226 PMC11939542

[23] Martin RD, Naziruddin Z. Systematic review of student anxiety and performance during objective structured clinical examinations. Currents Pharm Teach Learn. 2020;12:1491–7. 10.1016/j.cptl.2020.07.007. 33092780

[24] Huang S-S, Wu J-W, Huang C-C, Liang J-F, Yang Y-Y, Cheng H-M, et al. Examining the impact of stress, test anxiety, and general anxiety on academic performance among medical students: a systematic review and meta-analyses. Int J Health Promotion Educ. 2025;1–16. 10.1080/14635240.2025.2489382.

[25] Ansari S, Iqbal N. Prevalence of exam anxiety: A systematic review and meta-analysis. Can J Behav Sci /. 2025. 10.1037/cbs0000473. Revue canadienne des sciences du comportement.

[26] Page MJ, McKenzie JE, Bossuyt PM, Boutron I, Hoffmann TC, Mulrow CD, et al. The PRISMA 2020 statement: an updated guideline for reporting systematic reviews. BMJ. 2021;372:n71. 10.1136/bmj.n71. 33782057 PMC8005924

[27] Driscoll R. Westside Test Anxiety Scale Validation. Education Resources Information Center. 2007.

[28] Nist P, Diehl M, Test Anxiety Q. 1990. https://my.iecc.edu/files_user/LTCH/files/TestAnxiety.pdf. Accessed 26 Jul 2026.

[29] Ouzzani M, Hammady H, Fedorowicz Z, Elmagarmid A. Rayyan—a web and mobile app for systematic reviews. Syst Rev. 2016;5:210. 10.1186/s13643-016-0384-4. 27919275 PMC5139140

[30] Carra MC, Romandini P, Romandini M. Risk of bias evaluation of cross-sectional studies: adaptation of the Newcastle‐Ottawa scale. J Periodontal Res. 2025. 10.1111/jre.13405. PMC13665615 40293188

[31] R Core Team. R: A language and environment for statistical computing. Vienna, Austria: R Foundation for Statistical Computing; 2025.

[32] Balduzzi S, Rücker G, Schwarzer G. How to perform a meta-analysis with R: a practical tutorial. Evid Based Ment Health. 2019;22:153–60. 10.1136/ebmental-2019-300117. 31563865 PMC10231495

[33] Viechtbauer W. Conducting meta-analyses in R with the metafor package. J Stat Softw. 2010;36:1–48. 10.18637/jss.v036.i03.

[34] Rücker G, Schwarzer G, Carpenter JR, Schumacher M. Undue reliance on I(2) in assessing heterogeneity may mislead. BMC Med Res Methodol. 2008;8:79. 10.1186/1471-2288-8-79. 19036172 PMC2648991

[35] Borenstein M, Hedges LV, Higgins JPT, Rothstein HR. Introduction to meta-analysis. 2nd ed. Hoboken: Wiley; 2021.

[36] Bharti B, Malhotra N, Virk A, Kaur V, Malhi P, Loomba P. Peer-led mentoring intervention for test anxiety reduction among medical students: development, implementation, and prospective evaluation. BMC Med Educ. 2026;26:369. 10.1186/s12909-026-08697-8. 41622165 PMC12955027

[37] Darwish NE, Omar YM, Elsaadany MA, Essam M, Hamdy S, Shawky K, et al. Test anxiety among medical students in Egypt: prevalence, associated factors, and coping strategies - a national cross-sectional study. Res Square [Preprint]. 2026. 10.21203/rs.3.rs-8569897/v1.

[38] Mastour H, Ghalibaf AAM, Niroumand S. Remote online test anxiety during the coronavirus disease 2019 crisis: a cross-sectional study among medical students. Iran Red Crescent Med J. 2022;24(3):e1645. 10.32592/ircmj.2022.24.3.1645.

[39] Memon I, Omair A, Barradah OM, Almegren NM, Almuqbil MM, Batarfi OH, et al. Measurement of exam anxiety levels among medical students and their association with the influencing factors. Cureus. 2023;15(7):e41417. 10.7759/cureus.41417. 37546066 PMC10403227

[40] Mosavi Z, Khazaie H, Janatolmakan M, Rezaeian S, Khatony A. Effects of auricular acupressure on test anxiety in medical students: a randomized parallel-group trial. BMC Med Educ. 2023;23:835. 10.1186/s12909-023-04825-w. 37936159 PMC10629063

[41] Patel R, Varma J, Phatak A, Nimbalkar SM. Study of test anxiety amongst undergraduate medical students from the state of Gujarat. J Educ Health Promotion. 2023;12:325. 10.4103/jehp.jehp_1680_22. PMC10670926 38023085

[42] Sohail H, Hassan SM, Ali B, Irfan S, Siddiqui HF, Bansari K, et al. Impact of pre-exam anxiety on the academic performance of final year medical students. Preprints [Preprint]. 2020. 10.20944/preprints202010.0651.v1.

[43] Saravanan C, Kingston R. A randomized control study of psychological intervention to reduce anxiety, amotivation and psychological distress among medical students. J Res Med Sci. 2014;19:391–7. 25097619 PMC4116568

[44] Shaheen A, Azam F, Rabbani MW, Kazmi N. Correlation of computer-based test anxiety with medical students’ performance before, during and after assessments. Pak J Med Sci. 2022;38:476–80. 10.12669/pjms.38.3.4989. 35480517 PMC9002404

[45] Tata RS, Ramya B, Mopidevi V. Prevalence of test anxiety and their related coping strategies in medical students – a cross-sectional study. Archives Mental Health. 2024;25:67–71. 10.4103/amh.amh_48_23.

[46] Alshiblawy H, Oleiwi I, Alghuraibawi NA. Prevalence of pre-test anxiety among medical students in Iraqi medical colleges: insights into arterial blood pressure and its cerebral implications. Revista Latinoam De Hipertension. 2024;19(6). 10.5281/zenodo.12669188.

[47] Aziz N, Serafi AH. Increasing levels of test anxiety and psychological distress with advancing years of medical education. Br J Med Health Res. 2017;4:40–2.

[48] Jirjees F, Odeh M, Al-Haddad A, Ass’ad R, Hassanin Y, Al-Obaidi H, et al. Test anxiety and coping strategies among university students an exploratory study in the UAE. Sci Rep. 2024;14:25835. 10.1038/s41598-024-59739-4. 39468103 PMC11519940

[49] Khan AN, Rasool SA, Sultan A, Tahira I. Prevalence of examination related anxiety in a private medical college. J Ayub Med Coll Abbottabad. 2013;25:113–5. 25098071

[50] Sindhu BM, Kumbar AS, Shashank KJ, Rashmi BM. Study of examination-related anxiety levels and coping strategies adopted by undergraduate students at a medical college in Central Karnataka. Dentistry Med Res. 2023;11:21–5. 10.4103/dmr.dmr_26_23.

[51] Ali M, Asim H, Edhi AI, Hashmi MD, Khan MS, Naz F, et al. Does academic assessment system type affect levels of academic stress in medical students? A cross-sectional study from Pakistan. Med Educ Online. 2015;20:27706. 10.3402/meo.v20.27706. 26112353 PMC4481047

[52] Roy SK, Majumdar S, Mukherjee M, Paul A. Assessment of examination related anxiety among students in a medical college at Kolkata, India: a cross-sectional study. JCDR. 2022;16(10):LC40–3. 10.7860/JCDR/2022/58219.17079.

[53] Afzal H, Afzal S, Siddique SA, Naqvi SAA. Measures used by medical students to reduce test anxiety. J Pak Med Assoc. 2012;62:982–6. 23139995

[54] Arain FR. Does test anxiety and gender have an impact on OSCE Performance among medical students? MEWFM. 2021;20:101–7. 10.5742/MEWFM.2021.94165.

[55] Ćurčić B, Sokolović D, Radanović M, Pavlović D, Bjeletić M, Vuković D, et al. Test anxiety as a risk factor for use of psychoactive compounds in medical students. Biomedicinska istraživanja. 2024;15(2):1–9. 10.59137/BII202402406C.

[56] Farnia V, Mousavi SA, Parsamehr A, Alikhani M, Golshani S, Nooripour R, et al. The mediating role of emotional intelligence in coping strategies and test anxiety in students of Kermanshah University of Medical Sciences, Kermanshah, Iran in 2013–2014. Iran J Psychiatry Behav Sci. 2017;11(4):e9254. 10.5812/ijpbs.9254.

[57] Lin L, Hongyu Y, Xing J, Ying M, Zixuan L, Tingting L. The indirect associations of academic self-efficacy and brief fear of negative evaluation in the relationship between neurotic personality traits and test anxiety of medical sciences students. BMC Psychol. 2025;14:307. 10.1186/s40359-025-03790-x. 41413619 PMC12964671

[58] Rehman F, Saeed I, Khan N, Shahzad H, Janjua A, Ajmal Z. Measuring the level of examination anxiety among students in a private medical college in Lahore. Pak J Med Health Sci. 2018;12(3):1084–7.

[59] Sadaf S, Ishtiqa S, Jahan M, Farooq F, Mushtaque S, Sukhia D. Examination stress during COVID-19 and its coping interventions used by medical students of Pakistan. Pakistan Armed Forces Med J. 2022;72:1069–73. 10.51253/pafmj.v72i3.7035.

[60] Sharma A, Singh SP, Shekhar S, Kushwaha P, Gahlot A. Exam anxiety and its associated risk factors among Indian medical undergraduates. Natl J Community Med. 2023;14:348–56. 10.55489/njcm.140620232774.

[61] Song L, Xiao R, Wang C, Li C, Liu Q, Zhang Y, et al. Effect of group impromptu music therapy on improving test anxiety and emotional regulation ability in medical students. Front Psychol. 2024;15:1467830. 10.3389/fpsyg.2024.1467830. 39726618 PMC11670072

[62] Quek TTC, Tam WWS, Tran BX, Zhang M, Zhang Z, Ho CSH, et al. The global prevalence of anxiety among medical students: a meta-analysis. IJERPH. 2019;16:2735. 10.3390/ijerph16152735. 31370266 PMC6696211

[63] Omar YM, Khattab YA, Abdelmageed A, Messak M, Mandour Y, Shabeeb AE, et al. The impostor phenomenon construct in medical education: a meta-analysis of its prevalence and a critical appraisal of its measurement. Adv Health Sci Educ. 2026. 10.1007/s10459-026-10513-3. 41801596

[64] Lin Y-K, Saragih ID, Lin C-J, Liu H-L, Chen C-W, Yeh Y-S. Global prevalence of anxiety and depression among medical students during the COVID-19 pandemic: a systematic review and meta-analysis. BMC Psychol. 2024;12:338. 10.1186/s40359-024-01838-y. 38858700 PMC11163725

[65] Stankov L. Unforgiving Confucian culture: A breeding ground for high academic achievement, test anxiety and self-doubt? Learn Individual Differences. 2010;20:555–63. 10.1016/j.lindif.2010.05.003.

[66] Oswalt SB, Lederer AM, Chestnut-Steich K, Day C, Halbritter A, Ortiz D. Trends in college students’ mental health diagnoses and utilization of services, 2009–2015. J Am Coll Health. 2020;68:41–51. 10.1080/07448481.2018.1515748. 30355071

[67] Li W, Zhao Z, Chen D, Peng Y, Lu Z. Prevalence and associated factors of depression and anxiety symptoms among college students: a systematic review and meta-analysis. Child Psychol Psychiatry. 2022;63:1222–30. 10.1111/jcpp.13606. 35297041

