# Supplementary material for: Prevalence of test anxiety among medical students: a systematic review and meta-analysis
Source: BMC Med Educ. 2026 Sep 9;26:1448. doi: 10.1186/s12909-026-10270-2 (PMC13556011; doi:10.1186/s12909-026-10270-2)
Supplement: Supplementary file 1 [file 12909_2026_10270_MOESM1_ESM.docx]

**Part 1: Main Analysis Supplementary Figures & Tables**

**Table S1:** Search Strategy.

| **Database** | **Search strategy** | **Results** |
| --- | --- | --- |
| **PubMed** | ("Performance Anxiety"[Mesh] OR "test anxiety"[tiab] OR "test stress"[tiab] OR "exam anxiety"[tiab] OR "exam stress"[tiab] OR "pre-exam anxiety"[tiab] OR "pre exam anxiety"[tiab] OR "anticipatory anxiety"[tiab] OR "situational anxiety"[tiab] OR "evaluation anxiety"[tiab] OR "performance anxiety"[tiab]) AND ("Students, Medical"[Mesh] OR "medical student*"[tiab] OR "medical education"[tiab] OR "medical trainee*"[tiab] OR "medical school"[tiab] OR "medical undergraduate*"[tiab] OR "clinical student*"[tiab] OR "future physician*"[tiab] OR "physician trainee*"[tiab] OR "health profession student*"[tiab] OR "medical learner*"[tiab]) | 179 |
| **WOS** | ("test anxiety" OR "test stress" OR "exam anxiety" OR "exam stress" OR "pre-exam anxiety" OR "anticipatory anxiety" OR "situational anxiety" OR "evaluation anxiety" OR "pre exam anxiety" OR "performance anxiety") AND ("medical student*" OR "medical education" OR "medical trainee*" OR "medical school" OR "medical undergraduate*" OR "clinical student*" OR "future physician*" OR "physician trainee*" OR "health profession student*" OR "medical learner*") | 254 |
| **Scopus** | ("test anxiety" OR "test stress" OR "exam anxiety" OR "exam stress" OR "pre-exam anxiety" OR "anticipatory anxiety" OR "situational anxiety" OR "evaluation anxiety" OR "pre exam anxiety" OR "performance anxiety") AND ("medical student*" OR "medical education" OR "medical trainee*" OR "medical school" OR "medical undergraduate*" OR "clinical student*" OR "future physician*" OR "physician trainee*" OR "health profession student*" OR "medical learner*") | 348 |
| **Embase** | ("test anxiety" OR "test stress" OR "exam anxiety" OR "exam stress" OR "pre-exam anxiety" OR "anticipatory anxiety" OR "situational anxiety" OR "evaluation anxiety" OR "pre exam anxiety" OR "performance anxiety") AND ("medical student*" OR "medical education" OR "medical trainee*" OR "medical school" OR "medical undergraduate*" OR "clinical student*" OR "future physician*" OR "physician trainee*" OR "health profession student*" OR "medical learner*") | 320 |

**Table S2:** Test Anxiety Assessment Tools and Cutoff Criteria Used Across Included Studies.

| **Study ID** | **Tool used** | **Cutoff used** |
| --- | --- | --- |
| Afzal et al., 2012 | WTAS | Moderately high (3.0–3.4), high (3.5–3.9), and extremely high (4.0–5.0). |
| Ali et al., 2015 | WTAS | Moderately high (3.0–3.4), high (3.5–3.9), and extremely high (4.0–5.0). |
| Almutairi et al., 2024* | WTAS | Low (NR), Moderate (NR), High (NR) |
| Alshiblawy et al., 2024 | TAQ | Healthy test anxiety (20–35) and high (unhealthy) test anxiety (36–50). |
| Arain - 2021 | TAQ | Healthy test anxiety (20–35) and high (unhealthy) test anxiety (36–50). |
| Aziz and Serafi - 2017 | WTAS | Moderately high (3.0–3.4), high (3.5–3.9), and extremely high (4.0–5.0). |
| Bharti et al., 2026 | WTAS | Moderate-high (3.0–3.9) and severe (4.0–5.0). |
| Bischofsberger et al., 2021 | Prüfungsangstfragebogen (PAF) | Test anxiety (≥60). |
| Ćurčić et al., 2024 | WTAS | Medium (2.5–3.5), high (>3.5). |
| Darwish et al., 2025 | WTAS | Moderately high (30–34), high (35–39), and extremely high (40–50). |
| Farnia et al., 2017 | TAS | Moderate test anxiety (13–20) and high test anxiety (≥20). |
| Green et al., 2016 | WTAS | Test anxiety (≥3.0). |
| Jirjees et al., 2024 | WTAS | Moderately high (3.0–3.4), high (3.5–3.9), and extremely high (4.0–5.0). |
| Khan et al., 2013 | VAS | Moderate test anxiety (40-60) and severe test anxiety (70-100). |
| Lin et al., 2025* | TAS | Moderate test anxiety (13–20) and high test anxiety (≥20). |
| Liu et al., 2021 | TAS | Moderate test anxiety (13–20) and high test anxiety (≥20). |
| Mastour et al., 2022 | TAS | Moderate test anxiety (13–20) and high test anxiety (≥20). |
| Memon et al., 2023* | WTAS | Moderate test anxiety (2.6-3.4) and high test anxiety (3.5-5). |
| Mosavi et al., 2023 | TAS | Moderate test anxiety (13–20) and high test anxiety (≥20). |
| Patel et al., 2023 | WTAS | Moderately high (3.0–3.4), high (3.5–3.9), and extremely high (4.0–5.0). |
| Patil and Aithala, 2017 | WTAS | Moderately high (3.0–3.4), high (3.5–3.9), and extremely high (4.0–5.0). |
| Rehman et al., 2018 | VAS | Moderate test anxiety (40-60) and severe test anxiety (70-100). |
| Roy et al., 2022 | WTAS | Moderately high (3.0–3.4), high (3.5–3.9), and extremely high (4.0–5.0). |
| Rtbey et al, 2022 | WTAS | Moderately high (30–34), high (35–39), and extremely high (40–50). |
| Sadaf et al., 2022 | TAQ | Healthy test anxiety (20–35) and high (unhealthy) test anxiety (36–50). |
| Saravanan and Kingston, 2014 | WTAS | Moderately high (3.0–3.4), high (3.5–3.9), and extremely high (4.0–5.0). |
| Shaheen et al., 2022 | TAQ | Healthy test anxiety (20–35) and high (unhealthy) test anxiety (36–50). |
| Sharma et al., 2023 | WTAS | Moderately high (30–34), high (35–39), and extremely high (40–50). |
| Simran et al., 2015 | VAS | Moderate test anxiety (40-60) and severe test anxiety (70-100). |
| Sindhu et al., 2023 | WTAS | Moderately high (3.0–3.4), high (3.5–3.9), and extremely high (4.0–5.0). |
| Sohail et al., 2020 | WTAS | Moderately high (3.0–3.4), high (3.5–3.9), and extremely high (4.0–5.0). |
| Song et al., 2024 | TAS | High test anxiety (≥20). |
| Tata et al., 2024* | WTAS | Moderately high (30–34), high (35–39), and extremely high (40–50). |
| Tsegay et al., 2019 | WTAS | Test anxiety (≥30). |
| Zhang et al., 2022 | TAS | Moderate test anxiety (13–20) and high test anxiety (≥20). |
| TAQ – Test Anxiety Questionnaire; TAS – Test Anxiety Scale; WTAS – Westside Test Anxiety Scale; VAS – Visual Analogue Scale; PAF – Prüfungsangstfragebogen  *Combined low and moderate categories | | |

**Table S3:** Risk of Bias of the included studies using the simplified version of the Newcastle Ottawa Scale (NOS-xs2) for prevalence studies.

|  | **Risk of bias domains** | | |
| --- | --- | --- | --- |
|  | **Study sample selection** | | **Assessment of outcome** |
| **Study ID** | **1. Representativeness of the study sample** | **2. Sample size** | **3. Assessment of the outcome** |
| Afzal et al., 2012 | d) No description of the sampling strategy. | a) Justified. | b) Acceptable assessment tool. |
| Ali et al., 2015 | b) Somewhat representative of the target population (nonrandom sampling). | b) Not justified. | b) Acceptable assessment tool. |
| Almutairi et al., 2024 | b) Somewhat representative of the target population (nonrandom sampling). | b) Not justified. | b) Acceptable assessment tool. |
| Alshiblawy et al., 2024 | b) Somewhat representative of the target population (nonrandom sampling). | b) Not justified. | b) Acceptable assessment tool. |
| Arain - 2021 | b) Somewhat representative of the target population (nonrandom sampling). | a) Justified. | b) Acceptable assessment tool. |
| Aziz and Serafi - 2017 | a) Truly representative of the target population (all subjects). | b) Not justified. | b) Acceptable assessment tool. |
| Bharti et al., 2026 | a) Truly representative of the target population (all subjects). | a) Justified. | b) Acceptable assessment tool. |
| Bischofsberger et al., 2021 | b) Somewhat representative of the target population (nonrandom sampling). | b) Not justified. | b) Acceptable assessment tool. |
| Ćurčić et al., 2024 | a) Truly representative of the target population (all subjects). | b) Not justified. | b) Acceptable assessment tool. |
| Darwish et al., 2026 | b) Somewhat representative of the target population (nonrandom sampling). | a) Justified. | b) Acceptable assessment tool. |
| Farnia et al., 2017 | b) Somewhat representative of the target population (nonrandom sampling). | b) Not justified. | b) Acceptable assessment tool. |
| Green et al., 2016 | a) Truly representative of the target population (random sampling). | b) Not justified. | b) Acceptable assessment tool. |
| Jirjees et al., 2024 | b) Somewhat representative of the target population (nonrandom sampling). | b) Not justified. | b) Acceptable assessment tool. |
| Khan et al., 2013 | d) No description of the sampling strategy. | b) Not justified. | c) Non-acceptable assessment methods. |
| Lin et al., 2025 | b) Somewhat representative of the target population (nonrandom sampling). | a) Justified. | b) Acceptable assessment tool. |
| Liu et al., 2021 | a) Truly representative of the target population (random sampling). | a) Justified. | b) Acceptable assessment tool. |
| Mastour et al., 2022 | b) Somewhat representative of the target population (nonrandom sampling). | a) Justified. | b) Acceptable assessment tool. |
| Memon et al., 2023 | b) Somewhat representative of the target population (nonrandom sampling). | b) Not justified. | b) Acceptable assessment tool. |
| Mosavi et al., 2023 | b) Somewhat representative of the target population (nonrandom sampling). | a) Justified. | b) Acceptable assessment tool. |
| Patel et al., 2023 | b) Somewhat representative of the target population (nonrandom sampling). | b) Not justified. | b) Acceptable assessment tool. |
| Patil and Aithala, 2017 | d) No description of the sampling strategy. | b) Not justified. | b) Acceptable assessment tool. |
| Rehman et al., 2018 | b) Somewhat representative of the target population (nonrandom sampling). | b) Not justified. | c) Non-acceptable assessment methods. |
| Roy et al., 2022 | b) Somewhat representative of the target population (nonrandom sampling). | b) Not justified. | b) Acceptable assessment tool. |
| Rtbey et al, 2022 | a) Truly representative of the target population (random sampling). | a) Justified. | b) Acceptable assessment tool. |
| Sadaf et al., 2022 | b) Somewhat representative of the target population (nonrandom sampling). | b) Not justified. | b) Acceptable assessment tool. |
| Saravanan and Kingston, 2014 | d) No description of the sampling strategy. | b) Not justified. | b) Acceptable assessment tool. |
| Shaheen et al., 2022 | b) Somewhat representative of the target population (nonrandom sampling). | b) Not justified. | b) Acceptable assessment tool. |
| Sharma et al., 2023 | a) Truly representative of the target population (random sampling). | a) Justified. | b) Acceptable assessment tool. |
| Simran et al., 2015 | d) No description of the sampling strategy. | b) Not justified. | c) Non-acceptable assessment methods. |
| Sindhu et al., 2023 | a) Truly representative of the target population (all subjects). | a) Justified. | b) Acceptable assessment tool. |
| Sohail et al., 2020 | b) Somewhat representative of the target population (nonrandom sampling). | b) Not justified. | b) Acceptable assessment tool. |
| Song et al., 2024 | d) No description of the sampling strategy. | b) Not justified. | b) Acceptable assessment tool. |
| Tata et al., 2024 | a) Truly representative of the target population (random sampling). | a) Justified. | b) Acceptable assessment tool. |
| Tsegay et al., 2019 | a) Truly representative of the target population (random sampling). | a) Justified. | b) Acceptable assessment tool. |
| Zhang et al., 2022 | d) No description of the sampling strategy. | b) Not justified. | b) Acceptable assessment tool. |

**Figure S1:** Forest plot of the prevalence of test anxiety among medical students: Subgroup analysis by gender

**Figure S2:** Forest plot of the prevalence of test anxiety among medical students: Subgroup analysis by academic year

**Figure S3:** Forest plot of the prevalence of test anxiety among medical students: Subgroup analysis by region

**Figure S4:** Forest plot of the prevalence of test anxiety among medical students: Subgroup analysis by assessment tool

**Figure S5:** Forest plot of the prevalence of test anxiety among medical students: Subgroup analysis by administration timing

**Figure S6:** Forest plot of the prevalence of test anxiety among medical students: Subgroup analysis by sampling technique

**Table S4:** Meta-regression of data collection year on the prevalence of test anxiety among medical students

| **Term** | **Coefficient** | **95% CI** | **p-value** |
| --- | --- | --- | --- |
| Intercept | -5.800 | [-224.323, 212.723] | >0.9 |
| Data collection year | 0.003 | [-0.105, 0.111] | >0.9 |
| Studies = 35; F(1, 33) = 0.00, p = >0.9 | | | |
| Residual heterogeneity: Tau² = 1.3259, I² = 99.0% | | | |
| Coefficients and confidence intervals are on the Logit Transformed Proportion scale | | | |

**Figure S7:** Forest plot of the leave-one-out sensitivity analysis for the prevalence of test anxiety among medical students

**Part 2: Sensitivity Analysis Excluding VAS Studies**

**Figure S8:** Summary forest plot of subgroup analyses for the prevalence of test anxiety among medical students

 **Figure S9:** Forest plot of the prevalence of test anxiety among medical students

 **Figure S10:** Forest plot of the prevalence of test anxiety among medical students: Subgroup analysis by gender

 **Figure S11:** Forest plot of the prevalence of test anxiety among medical students: Subgroup analysis by academic year

 **Figure S12:** Forest plot of the prevalence of test anxiety among medical students: Subgroup analysis by region

 **Figure S13:** Forest plot of the prevalence of test anxiety among medical students: Subgroup analysis by assessment tool

**Figure S14:** Forest plot of the prevalence of test anxiety among medical students: Subgroup analysis by administration timing

 **Figure S15:** Forest plot of the prevalence of test anxiety among medical students: Subgroup analysis by sampling technique

**Table S5:** Meta-regression of data collection year on the prevalence of test anxiety among medical students

| **Term** | **Coefficient** | **95% CI** | **p-value** |
| --- | --- | --- | --- |
| Intercept | -131.744 | [-319.546, 56.057] | 0.2 |
| Data collection year | 0.065 | [-0.028, 0.158] | 0.2 |
| Studies = 32; F(1, 30) = 2.05, p = 0.2 | | | |
| Residual heterogeneity: Tau² = 0.8106, I² = 98.4% | | | |
| Coefficients and confidence intervals are on the Logit Transformed Proportion scale | | | |

 **Figure S16:** Bubble plot of meta-regression analysis showing the association between data collection year and the prevalence of test anxiety among medical students

 **Figure S17:** Forest plot of the leave-one-out sensitivity analysis for the prevalence of test anxiety among medical students

**Figure S18:** Forest plot of the prevalence of test anxiety among medical students in the sensitivity analysis including only severe/high test anxiety categories from studies that combined low and moderate anxiety levels into a single category
